# Reactivity of selected markers of innate and adaptive immunity in rabbits experimentally infected with antigenic variants of RHD (*Lagovirus europaeus*/GI.1a)

**DOI:** 10.1007/s11259-021-09851-x

**Published:** 2021-10-29

**Authors:** Paulina Niedźwiedzka-Rystwej, Beata Tokarz-Deptuła, Wiesław Deptuła

**Affiliations:** 1 Institute of Biology, University of Szczecin, Felczaka 3c, 71-412 Szczecin, Poland; 2 Institute of Veterinary Sciences, Faculty of Biological and Veterinary Sciences, Nicolaus Copernicus University in Toruń, Gagarina 7, 87-100 Toruń, Poland

**Keywords:** Rabbit, Viral infection, RHDV, RHDVa, *Lagovirus europeus*

## Abstract

*Lagovirus europaeus*/GI.1 causes a fatal viral condition in rabbits characterized by acute viral hepatitis and disseminated intravascular coagulation. Due to rapid viral and environmental changes variants (Lagovirus europaeus/GI.1a and GI.2) have appeared and few immunological studies were performed. The aim of the study was to determine innate and adaptive immunity parameters in rabbits infected with six *Lagovirus europeus*/GI.1a viruses. To achieve the goal several methods were used, i.e. cytometry, microscopy, biochemical and cytochemical tests, spectrophotometry. The results show that three immunotypes exists among the studied strains and they differ in innate (mainly) and adaptive immunity, partly depending on hemagglutination. The peak of changes is 24 h post infection in phagocytosis markers of polymorphonuclear cells and CD8^+^ T cells. *Lagovirus europaeus*/GI.1a strains differ from *Lagovirus europaeus*/GI.1 in terms of immunological response based on our previous work concerning the same parameters in immunological response against this disease.

## Introduction

The interest in *Lagovirus europaeus*/GI.1 that infects wild and domestic rabbits has been growing ever since the disease was identified in 1984 in China (Liu et al., 1984). This viral disease characterized by liver damage and disseminated intravascular coagulation is known as rabbit hemorrhagic disease (RHD), similar in etiology to acute viral hepatitis (Peacock et al., 2017; Rouco et al., 2018; Chen et al., 2018). RHD virus is found now world-wide and poses a threat mainly to rabbits in natural ecosystems (Monterroso et al., 2016; Rouco et al., 2018). Rabbits are an important part of human diet in Spain and Portugal (Abrantes et al., 2012b) and a significant part in the ecosystem (Wells et al., 2018). They are used as model laboratory animals (Esteves et al., 2019; Mage et al., 2018). Although extensive research on the biology of *Lagovirus europeus*/GI.1 has been conducted, it is hampered by the lack of an appropriate cell culture system for the virus (Abrantes et al., 2012b).

Immunological research has been primarily carried out only on viruses of *Lagovirus europeus*/GI.1 since 1997 in Poland and since 2008 in Portugal and Spain (Table 1). Some research was done in 1990s in China (Table 1). Research conducted on 36 different viruses of *Lagovirus europeus*/GI.1 of European origin with varied hemagglutination ability and pathogenicity showed that immunological factors play an important role in the pathogenesis of RHD (Nahurska et al., 2003; Hukowska-Szematowicz 2013; Niedźwiedzka-Rystwej and Deptuła 2010a; Niedźwiedzka-Rystwej 2013; Trzeciak-Ryczek et al., 2016; 2017). *Lagovirus europeus*/ GI.1 displays immunological diversity. Hence, *Lagovirus europaeus*/GI.1 was differentiated into immunotypes although it was not accounted for in the current taxonomy of the virus, as the new nomenclature is based on phylogenetic relationships (Le Pendu et al., 2017). As a ssRNA virus, it changes rapidly and antigenic variants of the virus (RHDVa Lagovirus europeus/GI.1a) were identified in 1996 in Italy (Capucci et al., 1998). A new variant of RHDV was identified in 2010 as RHDV2 (Le Gall-Recule & Zwingelstein 2011), later renamed as RHDVb (Dalton et al. 2015), now RHDV Lagovirus europeus/GI.2 (Le Pendu et al., 2017). RHDVa Lagovirus europeus/GI.1a strains show different ability to bind to monoclonal antibodies of the epitopes of VP60 protein (1H8 negative, 3B12 specific) (Capucci et al., 1998). It also has a characteristic structure of the hypervariable region E of the VP60 capsid protein, which contains antigenic determinant of 1H8 antibody (Capucci et al., 1998). Its property of antigenicity, i.e. its ability to bind to antibodies, was demonstrated (Niedźwiedzka-Rystwej and Deptuła 2010a), although earlier it hardly met the definition of an antigenic variant. The genome of *Lagovirus europeus*/GI.1a differs from that of *Lagovirus europeus*/GI.1 with amino acid substitutions in 344–370 positions of the VP60 protein, in C region (position 309 A → S), in E region (351 A → T, 359 A → G, 365 S → N, 369 N → A, 370 V → A, 386 G → N, 412 A → N, 416 A → T) and in F region (434 I → V) (Schirrmeier et al., 1999).

**Table 1:** Immunological studies on Lagovirus europeus GI.1

No	Methods			References
1	Phagocytosis of PMN cells	peripheral blood		Huang 1991*; Nahurska et al., 2003; Tokarz-Deptuła, 2009; Niedźwiedzka-Rystwej and Deptuła, 2010a; Hukowska-Szematowicz, 2013; Niedźwiedzka-Rystwej, 2013
2	B and T lymphocytes	peripheral blood		Huang, 1991*; Tokarz-Deptuła, 2009; Niedźwiedzka-Rystwej and Deptuła, 2010b; b; Hukowska-Szematowicz, 2013; Niedźwiedzka-Rystwej, 2013
		spleen		Teixeira et al. 2012
3	Apoptosis	granulocytes and lymphocytes of peripheral blood		Niedźwiedzka-Rystwej, 2013; Niedźwiedzka-Rystwej and Deptuła 2012a, b; Niedźwiedzka-Rystwej et al., 2013a, b
		hepatocytes		Jung et al., 2000**, Chen et al. 2018
4	Amount of cytokines, interleukines and growth factors	blood serum		Marques et al., 2012; Teixeira et al., 2012
		hepatocytes		Di Guardo 1991**
5	Molecular analysis/expression of genes coding chosen cytokines, interleukines, growth factors and TLR receptors	granulocytes and lymphocytes of peripheral blood	cytokines/ interleukins/growth factors	Sanchez-Campos et al., 2004; Garcia-Lastra et al., 2010; Trzeciak-Ryczek et al., 2015, 2016, 2017
			TLR 2,3,4	Trzeciak-Ryczek et al., 2015
		hepatocytes	cytokines/ interleukins/growth factors	Tunon et al., 2011; Crespo et al., 2016
			TLR 3,4,13	Tunon et al., 2011; Abrantes et al., 2012a; 2017; Crespo et al., 2016
6	IgG and IgM (RHDV2)	blood serum		Dalton et al. 2018

Legend: * - non-standard, currently not used methods ** - review papers

The current classification of *Lagovirus europeus*/GI.1 (Le Pendu et al., 2017), that accounts for its genetic variability, defines most RHDVa viruses from the former G6 genogroup (Gall-Recule et al., 2003) as GI.1a strains and RHDV from other genogroups as GI.1. Many studies (Capucci et al., 1998; Schirrmeier et al., 1999; Niedźwiedzka-Rystwej 2013) showed that *Lagovirus europaeus*/GI.1a compared to *Lagovirus europaeus*/GI.1 caused higher mortality in rabbits after a shorter incubation period. It is worth mentioning, that *Lagovirus europaeus*/GI.1a was never detected in natural conditions, it was always reported in rabbitries, even in countries of Iberian Peninsula, with very high circulation of *Lagovirus europaeus*/GI.1a (Abrantes et al., 2014). As a result, approximately a hundred strains of *Lagovirus europeus*/GI.1a are currently identified.

In order to understand the difference in immunological reactivity between *Lagovirus europeus*/GI.1 and *Lagovirus europeus*/GI.1a, we determined twelve innate immunity markers and five adaptive immunity markers in rabbits infected experimentally with four hemagglutinating and two non-hemagglutinating strains of RHDVa Lagovirus europaeus/GI.1a of different pathogenicity (Table 2).

**Table 2:** *Lagovirus europeus*/GI.1a used in the study

Name	GenBank Accession	Haema-gglutination ability	Mortality (hours post infection)	Pathotype	Innate immunity		Adaptive immunity	
					Ratio	Immunotype	Ratio	Immunotype
*Triptis*	EF558583	2560	100% (36 h)	I	0.17	II	0.23	II
*Hartmannsdorf*	EF558586	2560	100% (24 h)	I	0.12	II	0.16	III
*Vt97*	EU250331.1	2560	30% (36 h)	III	0.43	I	0.23	II
*72 V/2003*	MN218434.1	2560	100% (24 h)	I	0.17	II	0.20	II
*Pv97*	EU250330.2	0	100% (36 h)	I	0.40	I	0.10	III
*9905*	AJ302016.3	0	90%/36 h	I	0.08	III	0.30	I

Changes observed in immune response picture were to demonstrate existence of immunotypes (different immunogenicity) in six investigated viruses of *Lagovirus europeus*/GI.1a and to show a relation between their immunogenicity and their hemagglutination ability and between their immunogenicity and pathogenicity.

## Materials and methods

### Animal study

Tests were conducted on mixed-breed Polish rabbits weighing 3.2–4.2 kg, 6–8 months old, identified as conventional animals obtained from a licensed breeder (Anon., 1987). Rabbits were divided into six groups of ten animals. Each group was inoculated with one isolate of investigated *Lagovirus europaeus*/GI.1a collected 1996–2003 in different European countries (Table 2). Also recombinant in the capsid between *Lagovirus europaeus*/GI.1 and *Lagovirus europaeus*/GI.1a viruses were present among the samples (Abrantes et al., 2008; Forrester et al. 2009). Each group of investigated rabbits was accompanied by control groups that also consisted of ten animals. During experiments animals were kept in the vivarium at the Department of Microbiology and Immunology, Faculty of Biology, University of Szczecin. Temperature, humidity, light conditions, and cage sizes were adjusted to standards recommended in Poland and complying with Directive 2010/63/EU (Anon 2010). During the experiment the animals were constantly observed by the technician and research team members.

After animals had been transported to the vivarium, they underwent a two-week adaptation period. They were provided 0.15–0.20 kg of complete feed per day and had unlimited access to drinking water. Prior to experiments, no anti-RHDV antibodies were found in all subjects (commercial Mab ELISA kit (IZSLER, Italy). The study was approved by the Local Ethics Committee in Poznań, Poland (app.1/2006). All procedures involving animals were carried out in accordance with Polish legislation on animal welfare.

### Virus

Animals were challenged intramuscularly (lower limb muscle) administered *Lagovirus europeus*/GI.1a suspended in 1 ml of glycerol. Controls were given 1 ml of glycerol administered in the same way. Every examined viral strain sample was taken from naturally infected and dead rabbits from different European countries (Table 2), and livers were removed in post-mortem examinations. Twenty percent rabbit liver tissue homogenates in phosphate buffered saline were prepared and purified through centrifugation at 3000 rpm, 10% chloroforming for 60 min and another centrifugation interval. Liver homogenate samples were suspended in glycerol in 1:1 ratio (Niedźwiedzka-Rystwej and Deptuła, 2010a). All of the *Lagovirus europaeus*/GI.1a preparations contained the same number of viral particles as determined by density (1.34 g/dm3). Samples were stored in -80 °C for further use.

The hemagglutination ability was examined in hemagglutination assay using group 0 red blood cells. Pathogenicity was determined by recording clinical symptoms (in terms of body temperature and behavior) and mortality rate.

### Methods

#### Innate immunity

To estimate innate immunity in blood samples, twelve parameters were measured (Table 3): adherence ability of PMN (polymorphonuclear) cells and PMN absorption ability (absorption index and percentage of absorbing cells) was determined using methods described previously (Niedźwiedzka-Rystwej and Deptuła, 2010a). The ability to reduce Nitroblue-Tetrazolium (NBT) of PMN cells in peripheral blood was measured with cytochemical method in spontaneous, stimulated tests and spectrophotometric method. Additionally, determined parameters included: the coefficient of metabolic activity of granulocytes and the index of stimulation. The activity of myeloperoxidase (MPO) was determined histochemical staining. The concentration of lysozyme (LZM) in blood serum was determined with platelet diffusion method and LZM activity coefficient was determined. All methods were described before (Niedźwiedzka-Rystwej and Deptuła, 2010a).

**Table 3:** Methods used in the study

Innate immunity parameters
PMN Cell adherence capacity
Absorption Index
Percentage of absorbing cells
Spectrophotometric test for reduction of the Nitroblue-Tetrazolium (NBT)
Spontaneous test for reduction of the Nitroblue-Tetrazolium (NBT)
Stimulated test for reduction of the Nitroblue-Tetrazolium (NBT)
Stimulation index
Spontaneous metabolic activity coefficient of neutrophilic granulocytes
Stimulated metabolic activity coefficient of neutrophilic granulocytes
Activity of myeloperoxidase (MPO)
Concentration of lysozyme (LZM)
LZM activity index
Adaptive immunity parameters
Percentage of T lymphocytes (CD5 +)
Percentage of Th lymphocytes (CD4 +)
Percentage of Tc/Ts lymphocytes (CD8 +)
Percentage of T lymphocytes with CD25 + receptor
Percentage of B lymphocytes (CD19 +)

#### Adaptive immunity

Adaptive immunity was determined in blood by five indices (Table 3): CD4^+^ (Serotec, mouse anti rabbit—CD4, catalogue no. MCA799G), CD5^+^ (Serotec, mouse anti rabbit-CD5, catalogue no. MCA800), CD8^+^ (Serotec, mouse anti rabbit CD8, catalogue no. MCA1576G) and CD25^+^ (Serotec, mouse anti rabbit CD8, catalogue no. MCA1119GA) T cell count and by CD19^+^ B (Serotec, mouse anti-rabbit IgM B cell marker, catalogue no. MCA812GA), cell count using mouse anti-rabbit monoclonal antibody manufactured by Serotec, with a method earlier determined on a BD Facscanto II flow cytometer (Deptuła et al., 1998).

#### Sample collection

Blood samples were taken from the marginal vein of rabbit ear at zero hour, just prior to administration of viral antigen and/or glycerol (placebo). Then, samples were taken at 4, 8, 12, 24, 36 h post infection (hpi) or after manifestation of the first signs of disease, meeting the standards of the Local Ethics Committee.

Before the study the presence of anti-RHDV antibodies were checked with ELISA (commercial Mab ELISA kit (IZSLER, Italy), and after death of the animals the presence of RHD virus in liver was confirmed with real-time PCR (Light Cycler FastStart DNA Master SYBR Green I kit, Roche, Basel, Switzerland).

### Criteria of immunological assessment and pathogenicity determination of *Lagovirus europeus*/GI.1a viruses

Results obtained for the innate and adaptive immunity trials were analyzed with the Student t-test at the significance level set at p˂0.05 implemented in the software package Statistica 6.0.

Results of infected and control rabbits were compared in analysis of statistically significant increases and decreases that accounted for the number of investigated parameters, blood samples collected, number and character of (total) changes and number of examinations resulting from multiplying the number of parameters by the number of blood samples.

Because the number of collected blood samples was different for six investigated viruses of *Lagovirus europaeus*/GI.1a due to animal mortality, a ratio was calculated to define the strength of immune activity. The ratio was obtained by dividing the total number of changes in analyzed parameters by the number of examinations. The higher the value of this ratio, the greater the strength of the immune activity of the given virus of *Lagovirus europaeus*/ GI.1a. Thus, the ratio was the determinant of immunogenicity and immunotype of the examined virus of *Lagovirus europaeus*/GI.1a. It was assumed that values greater than or equal to 0.4 denoted high immunogenicity, values between 0.11 and 0.39 medium and values less than or equal to 0.1 low immunogenicity. Immunotype I was designated to those viruses with high immunogenicity, immunotype II was designated to those viruses with medium immunogenicity, and immunotype III was designated to those viruses with low immunogenicity.

Pathogenicity of investigated viruses of *Lagovirus europaeus*/GI.1a was determined based on mortality rate. It was classified according to criteria defined by Tokarz-Deptuła (2009) where pathotypes I, II and III denote strains causing 80–100%, 60–79% and below 60% mortality, respectively.

## Results

### Pathogenicity of the investigated *Lagovirus europaeus*/GI.1a viruses

Animals infected with 6 *Lagovirus europaeus*/GI.1a did not show any signs of the disease. The hemagglutinating recombinant Hartmannsdorf and 72 V/2003 caused 100% mortality at 24 hpi and hemagglutinating recombinant Triptis and non-hemagglutinating Pv97 at 36 h and all were classified as pathotype I. Non-hemagglutinating 9905, causing 90% mortality at 36 hpi was also classified as pathotype I. Hemagglutinating Vt97 caused 30% mortality at 36 h after infection and was classified as pathotype III (Table 2).

### Innate immunity results

**Fig. 1:**
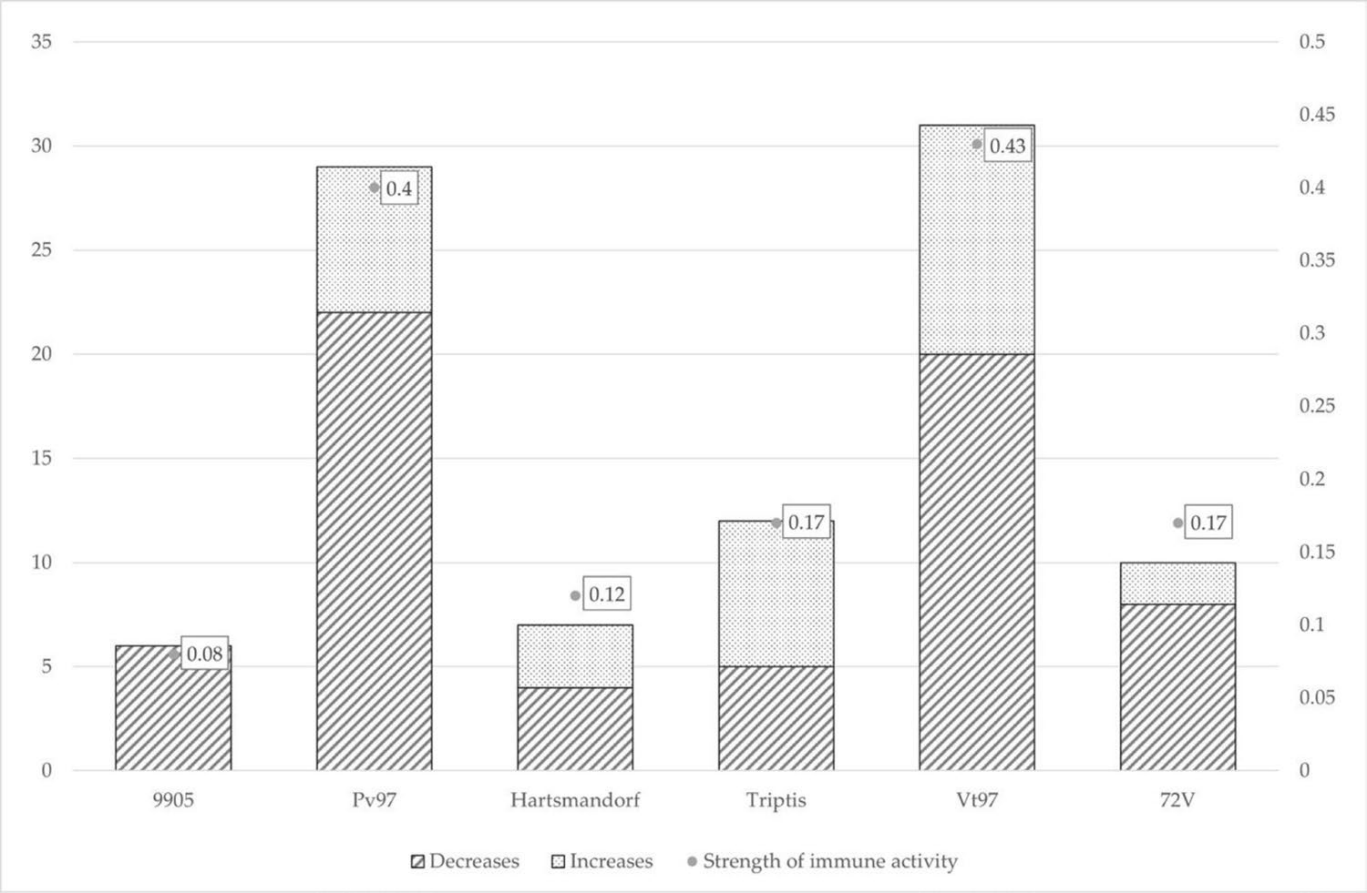
Strength of innate immune parameters activity of 6 *Lagovirus europaeus*/GI.1a

Results of innate immunity parameters showed that hemagglutinating (Triptis, Hartmannsdorf, Vt97, 72 V/2003 and non-hemagglutinating viruses (Pv97, 9905) of *Lagovirus europaeus*/ GI.1a triggered different responses and generated three immunotypes. The first immunotype is formed by the most immunogenic viruses—hemagglutinating Vt97 and non-hemagglutinating Pv97 with the ratio of the immune strength 0.43 and 0.40, respectively. The second immunotype are viruses of medium immunogenicity—hemagglutinating Triptis and 72 V/2003 of the same ratio of the immune strength 0.17 and hemagglutinating Hartmannsdorf with the ratio of the immune strength 0.12. The third immunotype with low immunogenicity is formed by non-hemagglutinating 9905 with the ratio of the immune strength 0.08 (Fig. 1).

Investigation into the impact of hemagglutionation capacity of *Lagovirus europaeus*/GI.1a on their immunogenicity did not give a conclusive relation. Out of four hemagglutinating viruses (Triptis, Hartmanndorf, Vt97, 72 V/2003) Vt97 was classified as immunotype I, Triptis, Vt97 and 72 V/2003 strains as immunotype II and the two non-hemagglutinating Pv97 and 9905 as immunotypes I and III, respectively.

There was even less of a trend between the immunotype and the pathotype of the six investigated *Lagovirus europeus*/GI.1a strains. Five *Lagovirus europeus*/GI.1a viruses (Triptis, Hartmannsdorf, 72 V/2003, Pv97, 9905) with high pathogenicity belonged to pathotype I which was not in compatibility with their immunogenicity. Vt97 classified in the group of the most immunogenic strains, it was characterized with the lowest pathogenicity of only 30% and belonged to pathotype III.

Analysis of changes in innate immunity to the *Lagovirus europaeus*/GI.1a showed primary decreases of investigated parameters and formed three immunotypes (I, II, III) and two pathotypes (I, III). Out of ninety five observed changes, sixty five were decreases and only thirty increases. The most significant increases were found in adherence ability of PMN cells and in percentage of absorbing cells.

Regardless of immunotype (immunogenicity), hemagglutination ability and pathotype of six investigated *Lagovirus europaeus*/GI.1a strains, decreases were observed mainly at 8 and 12 hpi and shortly before death. The longest-lasting changes were observed for non-hemagglutinating Pv97 and hemagglutinating Vt97. Interestingly, recombinant viruses – Tripts and Hartmannsdorf formed the same immunotype and pathotype. Most changes, about 20%, including increases and decreases, were observed at 24 hpi. It may indicate that 24 hpi is crucial in the course of infection regardless of hemagglutination capacity, immunogenicity and pathogenicity of all investigated *Lagovirus europaeus*/GI.1a.

### Adaptive immunity results

Analysis of five investigated adaptive immunity parameters in rabbits infected with six *Lagovirus europaeus*/GI.1a viruses, showed different, but less intense response. The ratio of the immune strength, varied between 0.10 and 0.30, compared to 0.08—0.43 for innate immunity. Regardless of these facts, adaptive immunity results showed that the viruses also formed 3 immunotypes – immunotype I with 9905 (ratio of the immune strength 0.30); immunotype II Triptis and Vt97 (ratio of the immune strength 0.23) and 72 V/2003 (0.20) and immunotype III with Hartmannsdorf (0.16) and Pv97 (0.10).

**Fig. 2:**
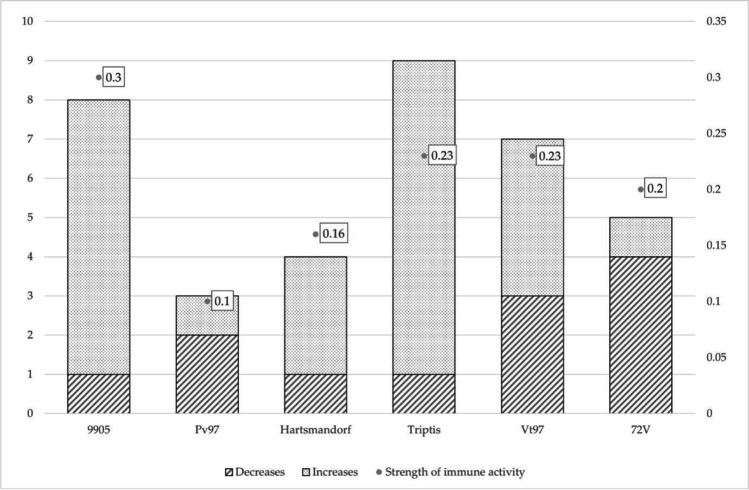
Strength of adaptive immune parameters activity of 6 *Lagovirus europaeus*/GI.1a

Investigation into the effect of hemagglutination capacity of six *Lagovirus europeus*/GI.1a on their immunogenicity showed that the effect was less significant than that of innate immunity parameters (Fig. 2).

The trend between immunogenicity and pathogenicity of *Lagovirus europaeus*/GI.1a in adaptive immunity was minimal and also no similar pattern was observed as far as recombinant viruses are concerned.

Assessment of the character of changes in adaptive immunity of six investigated *Lagovirus europeus*/GI.1a showed that adaptive immunity parameters were significantly less reactive than those of innate immunity. Only 36 changes were observed, i.e. almost 30% less than for innate immunity with 95 observed changes. Most changes were increases. The decreases were most intensive in the percentage of CD8 + T cells. Adaptive immunity changes were evenly distributed over the course of infection as they occurred between 4 and 36 h after infection. The time of their occurrence was not dependent on hemagglutination capacity, immunogenicity or pathotype of the investigated strains.

## Discussion

### Pathogenicity

The very high mortality rate of 90–100% at 24–36 h (Table 2) of animals infected with five investigated *Lagovirus europaeus*/GI.1a (Triptis, Hartmannsdorf, Pv97, 9905 and 72 V/2003) viruses confirms earlier observations of *Lagovirus europaeus*/GI.1a that *Lagovirus europaeus*/GI.1a has greater pathogenic activity than previous *Lagovirus europaeus*/GI.1 viruses (Capucci et al., 1998; Schirrmeier et al., 1999; Niedźwiedzka-Rystwej, 2013). It is worth noting, that currently *Lagovirus europaeus*/GI.1 and *Lagovirus europaeus*/GI.1a is being replaced by *Lagovirus europeus*/GI.2 (Rouco et al., 2018). Vt97 with Italian origin turned out to be an exception in the group of *Lagovirus europaeus*/GI.1a investigated in the present study, causing only 30% mortality at 36 hpi. The reason for this may be either difference in the virulence of the virus or the individual susceptibility differences. A similar mortality rate in the group of *Lagovirus europaeus*/GI.1 was observed for the Czech V-558 virus, which caused 40% mortality at 36 h (Hukowska-Szematowicz, 2013).

### Innate immunity

Results of immune response to six investigated *Lagovirus europaeus*/GI.1a caused the division of the viruses into three immunotypes, confirming findings reported by Tokarz-Deptuła (2009) who determined immunogenicity of ten *Lagovirus europaeus*/GI.1 from France and Poland. A similar division into three immunotypes based on the same parameters of innate immunity was made by Hukowska-Szematowicz (2013) in a study on sixteen *Lagovirus europaeus*/GI.1 viruses from different European countries and by Niedźwiedzka-Rystwej (Niedźwiedzka-Rystwej and Deptuła, 2010a) in a study on ten European *Lagovirus europeus*/GI.1 strains with varied ability to hemagglutination. So far, no immunotype differentiation has been performed as far as recombinant strains are concerned.

Hemagglutination capacity of six *Lagovirus europeus*/GI.1a viruses had inconclusive effect on their immunogenicity. The study showed that immunotype I was formed not only by hemagglutinating but also by non-hemagglutinating viruses. This is partly consistent with observations made by Tokarz-Deptuła (2009) who demonstrated that non-hemagglutinating *Lagovirus europaeus*/GI.1 and those of varied ability for hemagglutination formed immunotypes II and III. In contrast, another study of Nahurska et al. (2003) showed that non-hemagglutinating viruses were characterized by different immunogenicity compared to hemagglutinating ones.

Limited link between immunogenicity and pathogenicity of the *Lagovirus europaeus*/GI.1a viruses found in the present study is partly consistent with findings reported by Hukowska-Szematowicz (2013) who studied 12 *Lagovirus europaeus*/GI.1 viruses and found that pathogenicity correlated with immunogenicity to a medium degree only. That finding was not confirmed by Tokarz-Deptuła (2009) who observed that pathotype of the investigated *Lagovirus europaeus*/GI.1 was closely correlated with immunotype, particularly in case of very immunogenic viruses (immunotype I).

Regarding changes of innate immunity parameters given by increases and decreases, *Lagovirus europaeus*/GI.1a antigenic variants displayed mainly decreases. That finding would be consistent with results obtained for thirty *Lagovirus europaeus*/GI.1 of varied hemagglutination capacity (Nahurska et al., 2003; Tokarz-Deptuła, 2009; Hukowska-Szematowicz, 2013; Niedźwiedzka-Rystwej and Deptuła, 2010a, b; Niedźwiedzka-Rystwej, 2013). The reason for fewer changes observed for *Lagovirus europaeus*/GI.1a in the present study compared to mainly French and Polish *Lagovirus europaeus*/GI.1 may be due to the duration of disease. Most *Lagovirus europaeus*/GI.1 viruses caused mortality at 60 hpi while *Lagovirus europaeus*/GI.1a in the present study caused mortality at 36 hpi. In the study on thirty *Lagovirus europeus*/GI.1 viruses, changes were observed at 8–12 h, 24–36 h and 52–60 hpi, while for *Lagovirus europaeus*/GI.1a 24 hpi was particularly important. Interestingly, in *Lagovirus europaeus*/GI.1 viruses changes in immune response picture were also observed later (24–36 hpi), which was not the case for *Lagovirus europaeus*/ GI.1a.

The present study found that reactivity of immunity markers, mainly decreases of adherence ability and ability to absorb PMN cells, is consistent with results obtained for rabbits infected with Polish strains of *Lagovirus europaeus*/GI.1 with varied hemagglutination capacity (Tokarz-Deptuła, 2009; Niedźwiedzka-Rystwej, 2013), where the highest variation was observed as decreases of adherence ability of PMN cells. It was confirmed in a study on European *Lagovirus europeus*/ GI.1 viruses (Hukowska-Szematowicz, 2013), where the highest reactivity was found in phagocytosis defined as oxygen-related cidal activity.

Innate immunity markers in rabbits infected with *Lagovirus europaeus*/GI.1a, defining phagocytosis and particularly ability of PMN adherence and absorption, confirm the role of neutrophils in viral infection (Elbim et al., 2009). Lifespan of neutrophils contributes to PMN’s pathogen killing capacity and the immune reactivity triggers as soon as 4 and 8 hpi. contributes to effective fight against *Lagovirus europaeus*, but this infection is too rapid and extensive for neutrophils to win.

### Adaptive immunity results

Analysis of adaptive immunity to *Lagovirus europaeus*/GI.1a showed immunological diversity, although it was substantially to a smaller extent than innate immunity, but also showed the existence of three immunotypes, which is consistent with a previous study considering ten *Lagovirus europaeus*/GI.1 strains from different European countries with varied hemagglutination capacity (Niedźwiedzka-Rystwej, 2013). Results regarding the relation between immunogenicity and hemagglutination capacity of the *Lagovirus europaeus*/GI.1a are not consistent with the findings of Niedźwiedzka-Rystwej and Deptuła (2010b) for three non-hemagglutinating *Lagovirus europaeus*/GI.1 viruses which formed only two immunotypes (I and II). A study on one *Lagovirus europaeus*/GI.1 with varied hemagglutination found that the picture of immunological responses in terms of adaptive response markers was very different from that of the earlier examined sixteen HA + *Lagovirus europaeus*/GI.1 viruses (Niedźwiedzka-Rystwej and Deptuła, 2012a, b). No studies conducted to date demonstrated a connection between ability to hemagglutination and immunogenicity in *Lagovirus europaeus*/GI.1.

The lack of relation between immunogenicity and pathogenicity of six *Lagovirus europaeus* /GI.1a was not consistent with a study on ten *Lagovirus europeus*/GI.1 viruses with varied ability for hemagglutination, where a relation between pathotype I and immunotype I was observed (Tokarz-Deptuła, 2009). The study on 16 *Lagovirus europaeus*/GI.1 with varied ability of hemagglutination from different European countries (Hukowska-Szematowicz, 2013) did not find the link.

Adaptive immunity results of rabbits infected with *Lagovirus europaeus*/GI.1a are not consistent with findings obtained earlier in a study on *Lagovirus europaeus*/GI.1 with varied ability for hemagglutination. The present study found mainly increases of the investigated markers while others confirmed decreases. It is possible that these differences can be due to the duration of disease, i.e. 60–72 h for *Lagovirus europaeus*/GI.1 and 24–36 h for *Lagovirus europaeus*/GI.1a. The percentage of CD8 + T cells was the most reactive marker for *Lagovirus europaeus*/GI.1a. By contrast, the percentage of CD4^+^ T cells was the most reactive marker in case of *Lagovirus europaeus*/GI.1 (Tokarz-Deptuła, 2009; Hukowska-Szematowicz 2013; Niedźwiedzka-Rystwej, 2013). Changes of adaptive immunity markers in CD8^+^ T cells in rabbits infected with *Lagovirus europaeus*/GI.1a provide evidence for T cell acting as a link between innate and adaptive immunity. It is confirmed that viral antigen displaying neutrophils are an important cell type capable of acting as antigen presenting cell (APC) for effector CD8^+^ T lymphocytes (Hufford et al., 2012). This seems to be the case in rabbits infected with antigenic variants of RHDV, where statistically significant increase of CD8^+^ T cells was observed.

## Conclusion

The investigated six *Lagovirus europaeus*/GI.1a showed different immunogenicity, primarily in innate immunity, and detected existence of three immunotypes. Contrary to *Lagovirus europaeus*/GI.1, the immunotypes are mainly determined by hemagglutination capacity and to a smaller degree by pathogenicity. Recombinants seem to be very similar as far as innate immunity and pathogenicity are concerned and differ in the adaptive immunity. Immunological changes triggered by the activity of 6 investigated *Lagovirus europaeus*/GI.1a viruses are different from those of *Lagovirus europaeus*/GI.1 in terms of duration (from 4–8 h to 36 h with a peak at 24 h) and character. They are primarily observed in phagocytosis markers of PMN cells and CD8^+^ T cells.

## Data Availability

Do not apply.
